# Novel Osteogenic Ti-6Al-4V Device For Restoration Of Dental Function In Patients With Large Bone Deficiencies: Design, Development And Implementation

**DOI:** 10.1038/srep20493

**Published:** 2016-02-08

**Authors:** D. J. Cohen, A. Cheng, A. Kahn, M. Aviram, A. J. Whitehead, S. L. Hyzy, R. M. Clohessy, B. D. Boyan, Z. Schwartz

**Affiliations:** 1 Department of Biomedical Engineering, Virginia Commonwealth University, Richmond, VA, U.S.A; 2 Wallace H. Coulter Department of Biomedical Engineering, Georgia Institute of Technology and Emory University, Atlanta, GA, U.S.A; 3 Department of Biomedical Engineering, Peking University, Beijing, China; 4 Department of Oral and Maxillofacial Surgery, Tel Aviv University, Tel Aviv, Israel; 5 Tipul Behiuch Private Clinic, Tel Aviv, Israel; 6 Department of Periodontics, University of Texas Health Science Center at San Antonio, San Antonio, TX, U.S.A

## Abstract

Custom devices supporting bone regeneration and implant placement are needed for edentulous patients with large mandibular deficiencies where endosteal implantation is not possible. We developed a novel subperiosteal titanium-aluminum-vanadium bone onlay device produced by additive manufacturing (AM) and post-fabrication osteogenic micro-/nano-scale surface texture modification. Human osteoblasts produced osteogenic and angiogenic factors when grown on laser-sintered nano-/micro-textured surfaces compared to smooth surfaces. Surface-processed constructs caused higher bone-to-implant contact, vertical bone growth into disk pores (microCT and histomorphometry), and mechanical pull-out force at 5 and 10 w on rat calvaria compared to non surface-modified constructs, even when pre-treating the bone to stimulate osteogenesis. Surface-modified wrap-implants placed around rabbit tibias osseointegrated by 6 w. Finally, patient-specific constructs designed to support dental implants produced via AM and surface-processing were implanted on edentulous mandibular bone. 3 and 8 month post-operative images showed new bone formation and osseointegration of the device and indicated stability of the dental implants.

Currently, 23% of American adults over the age of 65 are completely edentulous^1^, and 37.9 million adults in the United States will have no natural teeth by 2020. Although the number of edentulous adults is expected to decrease by 10%, this is overshadowed by the 79% increase in the adult population over the age of 55^2^. Implant supported dentures significantly improve the quality of life in comparison to removable dentures^3^, but many of these individuals have significant bone loss, which may be unsuitable for implant placement.

A number of strategies have been used to enable implant placement when there is insufficient bone to provide stability for individual implants. Subperiosteal implants that follow the contours of the bony ridge of the jaw have had low success rates due to their failure to osseointegrate with the bone^4^. Approaches using dentures, partial dentures, or an implant supported bridge can provide a compromise solution to restore functional dentition. In many cases, a bone regeneration strategy using various bone graft materials is used to restore bone volume prior to the placement of the implant. This requires an additional procedure, in some cases involving the use of a membrane to guide the regenerating tissues, and complications may result^5^,^6^. However, in some cases, treatment using current options is not possible, particularly when the mental nerve is exposed. In these situations, a patient-specific strategy that stimulates bone regeneration to restore ridge height, protect any exposed nerve, and stabilize the device via osseointegration is needed in order to provide adequate support for rehabilitation of the dentition.

Our approach was to develop a one-step custom device that could be placed subperiosteally on the bone surface and by its osteogenic surface properties generate new bone, thereby becoming osseointegrated. Additive manufacturing (AM) provides a powerful method for fabricating three-dimensional (3D) metal devices based on computerized tomography (CT) of individual patients, enabling optimal fit between the implant and the contours of the patient’s existing bone. To address the goal of stimulating sufficient new bone to stabilize the device via osseointegration and ultimately to support reconstruction of the dentition, we took advantage of *in vitro* and *in vivo* observations using solid titanium (Ti) and titanium-aluminum-vanadium (Ti-6Al-4V) implants manufactured via conventional machining technology followed by grit blasting and acid etching. These studies showed that osteoblast differentiation and maturation were increased when osteoprogenitor cells were cultured on surfaces with microscale and nanoscale roughness compared to smooth surfaces^7^,^8^,^9^,^10^. Moreover, preclinical and clinical studies showed that peri-implant osteogenesis was enhanced when the surface had microscale and nanoscale roughness^11^,^12^,^13^,^14^,^15^. Similarly, microscale roughness on 3D nanofiber mesh surfaces supported greater osteoblastic differentiation of human mesenchymal stem cells (MSCs) *in vitro*^16^.

Recently, we showed that 3D Ti-6Al-4V constructs could be generated by AM and then processed via grit blasting and acid etching to have microscale and nanoscale roughness^17^.Osteoblast-like MG63 cells exhibited differentiation in a porosity and surface-roughness dependent manner when cultured on these constructs with hierarchical surface roughness. Taken together with previous studies using conventionally manufactured Ti and Ti-6Al-4V disks and implants described above, these results suggested that a micro-/nano-textured device could be designed that would stimulate bone regeneration sufficient to support rehabilitation of the dentition in an edentulous patient.

We used a step-wise approach to test this hypothesis (Fig. S1). To verify that osteoblast differentiation was sensitive to surface micro-/nano-topography, we assessed the response of normal human osteoblasts (NHOst cells) to the surface of surface modified Ti-6Al-4 V constructs as a function of osteogenic factor production. A rat cranial bone onlay model was used to analyze osseointegration of implants with a macroporous design to enhance osseointegration. We tested device osseointegration using several clinical scenarios involving pre-treatment of the calvarial bone to stimulate osteogenesis, including decalcification of the bone surface and use of a demineralized bone matrix putty (DBX). Next, implant osseointegration of surface-processed AM-fabricated custom wrap implants in a rabbit tibial bone onlay model was examined. Finally, two clinical case studies are presented that highlight the use of customized devices produced via AM and post-fabrication surface modification in edentulous patients. 3D Ti-6Al-4 V constructs were fabricated by laser sintering based on computed tomography (CT) scans of the patients’ mandibles and processed to create micro-/nano-textured surfaces. Follow-up radiographs at 3 and 8 months post-surgery demonstrate the successful osseointegration of the device and support of dental implants.

## Results

### Osteoblast response was enhanced on laser-sintered constructs with microscale and nanoscale surface roughness

We previously showed that additive manufacturing via laser sintering could be used to fabricate solid Ti-6Al-4V disks^17^. After modifying the surface by grit blasting and acid etching, the resulting texture had both microscale and nanoscale roughness, and was hydrophilic. We continued using these manufacturing and post-processing methods to create materials for this study.

**Figure 1:**
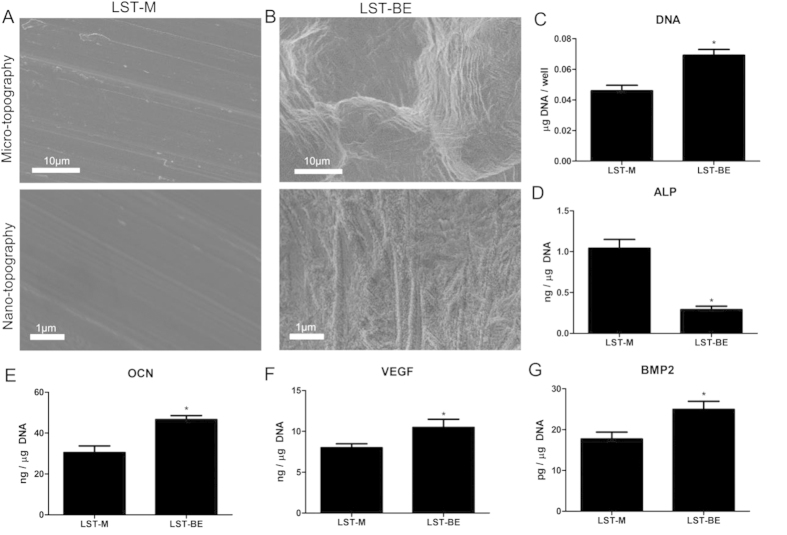
Cellular response to laser sintered disks with smooth or rough surfaces. Laser sintered surfaces were polished (**A**) or treated by blasting with calcium phosphate particles and subsequent acid etching (**B**) that resulted in combined micro- (top) and nano-roughness (bottom). DNA content (**C**), osteocalcin (**D**), osteoprotegerin (**E**), VEGF (**F**), and BMP2 (**G**) production by NHOst cells seeded on disks. Student’s t-test, p < 0.05, *vs. LST-M.

We first examined the response of normal human osteoblasts (NHOst cells) to the surface using 2D Ti-6Al-4V disks that were produced by laser sintering using the same methods as previously described^17^. The Ti-6Al-4V disks had one of two surface topographies: polished surfaces had a relatively smooth micro- and nano-topography (LST-M) (Fig. 1A) compared to grit blasted and acid etched surfaces (LST-BE), which possessed both micro- and nano-roughness (Fig. 1B). More NHOst cells were present on the LST-BE surfaces based on DNA content of the cultures (Fig. 1C), but cultures on LST-BE had lower alkaline phosphatase specific activity (Fig. 1D) than cells grown on LST-M surfaces. Cells on the rougher LST-BE surfaces produced more osteocalcin (Fig. 1E), bone morphogenetic protein 2 (BMP2) (Fig. 1F), and vascular endothelial growth factor A (VEGF-A) (Fig. 1G) than NHOst cells on LST-M.

### Surface treatment of calvaria did not affect bone growth into porous constructs

An initial rat cranial bone onlay study was performed to determine if osseointegration of the device would be enhanced by inclusion of through pores to facilitate migration of host osteoprogenitor cells and to increase surface area to allow for vertical bone growth from the calvarial surface through the implant. 5 mm diameter disks with twelve 0.5 mm diameter holes were laser sintered followed by grit blasting and acid etching in the manner previously described^17^. Clinically, etching treatments may be used on bone prior to implant placement to increase surface area, expose more bone morphogenetic protein stored in the extracellular matrix, and increase the availability of mesenchymal cells for improved osseointegration^18^,^19^. Therefore, we also assessed whether pretreatment of the calvarial bone by etching would alter the extent of new bone formation. We treated male Sprague-Dawley rat calvarial bone surfaces prior to implant placement with one of two methods and examined osseointegration at 5 and 10 weeks. After elevating the periosteum, the calvarial bone cortex was perforated 15–20 times with a dental burr to expose bone marrow derived stem cells to the implant, as would be done clinically. Calvaria (6 rats per treatment group) were treated with 24% ethylene diamine tetra acetic acid (EDTA), 37% phosphoric acid, or were left untreated after perforation. The disks were then secured to the underlying bone by approximating the periosteum to the implant via resorbable suture (Fig. S2a).

MicroCT analysis revealed that the pores did improve osseointegration, but pre-treatment of the calvaria had no effect. Bone-to-implant contact (Fig. S2B) and bone in-growth (Fig. S2C) in the disk pores were not significantly different across groups at both 5 and 10 weeks after implantation. Histology supported the microCT observations at 5 and 10 weeks (Fig. S2D). Interestingly, implants were not only osseointegrated with the calvaria, but new bone nodules were observed growing through the pores. Bone-to-implant contact determined by histomorphometry (Fig. S2E) confirmed the microCT results. Bone in-growth analyzed histomorphometrically (Fig. S2F) was higher than the microCT values, but the results still did not identify differences among treatment groups. Because analysis via microCT and histology showed no significant differences between calvarial bone growth into implants with pre-implantation treatment, no treatment was used for future studies.

### Calvarial bone formation in constructs was increased in the presence of demineralized bone matrix

Demineralized bone matrix (DBM) is commonly used in clinical cases where bone regeneration is needed prior to implant placement. To determine whether inclusion of DBM would impact the osteogenic capacity of the implant design, we compared bone formation using laser sintered disks in the presence and absence of human DBM putty in hyaluronic acid (DBX^®^, Musculoskeletal Transplant Foundation, Edison, NJ). For these experiments, the disks were treated by grit blasting and acid etching to have the microscale and nanoscale roughness as well as the hydrophilicity described above. After elevating the periosteum, the calvarial bone cortex was perforated using a dental bur. In one-half of the athymic nude rats, the calvarial bone surface was coated with DBX prior to implant placement. After placing these implants, the implant surface was also coated with DBX. The periosteum and skin were then restored.

**Figure 2:**
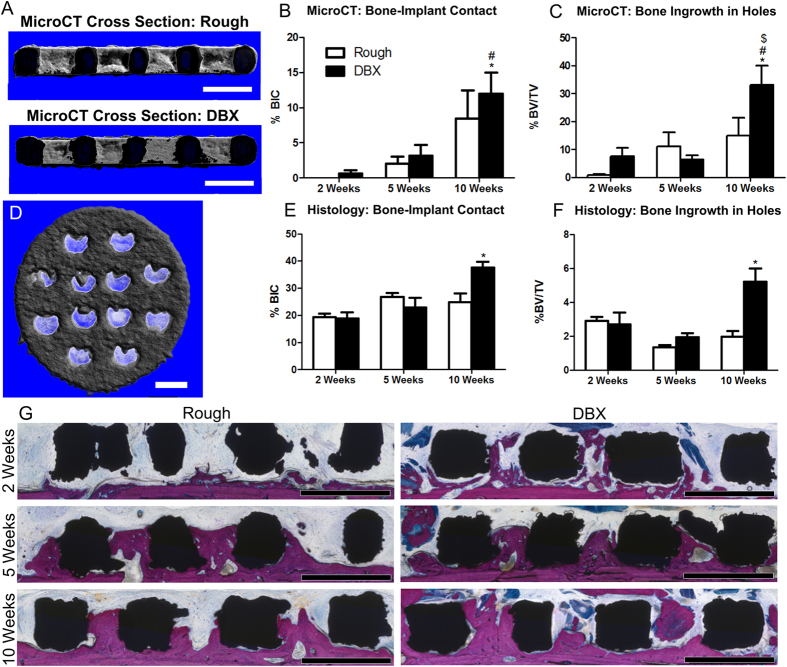
The effect of demineralized bone matrix on bone growth into porous disks. Disks were implanted on calvaria of athymic nude rats with and without DBX, and bone-to-implant contact was assessed after 2, 5, and 10 weeks. MicroCT cross-sectional images show sintered disks ((**A**), rough) implanted on calvaria of rats without ((**A)**, rough) or with ((**A**), DBX) demineralized bone matrix after 10 weeks, which black indicating the implant and white indicating new bone formation. These images were analyzed for bone-to-implant contact (**B**) and bone ingrowth in holes (**C**). MicroCT top down images, thresholded to remove underlying calvaria, revealed new bone growth into pores of the disk (**D**). Histomorphometric analysis was conducted per implant to evaluate bone-to-implant contact (**E**) and bone ingrowth in holes (**F**). Histological images depict bone growth along sides of disk pores for both groups (**G**). MicroCT and histological image scale bars represent 1 mm. One-way ANOVA with Bonferroni analysis, p < 0.05, * vs. all groups (**B,C**), * vs. 2 week rough, # vs. 2 week DBX, $ vs. 5 week DBX (**E,F**).

At 2, 4, and 10 weeks post-operatively, microCT cross-sectional analysis of the entire implant was performed to assess bone-to-implant contact and bone ingrowth in holes (Fig. 2A). Bone-to-implant contact analyzed by microCT was not significantly different between untreated and DBX-treated calvaria at 2 and 5 weeks, but DBX-treated sites had significantly higher bone-to-implant contact at 10 weeks when compared to both non-treated and DBX-treated sites at all time points (Fig. 2B). Bone in growth in implants holes supported the microCT observations, with DBX-treated sites also having a higher percent age of bone in-growth at 10 weeks when compared to non-treated and DBX-treated sites at all of the time points (Fig. 2C). Top down microCT images also confirmed bone growth into implant pores (Fig. 2D). Bone-to-implant contact determined histologically was increased in DBX-treated sites at 10 weeks compared to both non-treated and DBX-treated sites at 2 weeks (Fig. 2E). Percent bone in-growth in holes was increased in DBX-treated sites at 10 weeks compared to non-treated and DBX-treated sites at 2 weeks, and DBX-treated sites at 5 weeks (Fig. 2F). Histological sections (Fig. 2G) revealed bone growth from the bottom of non-treated sites while bone growth was achieved from both the bottom and the top of DBX-treated sites.

### Surface roughness and DBX enhanced mechanical integration of bone with porous implants

**Figure 3:**
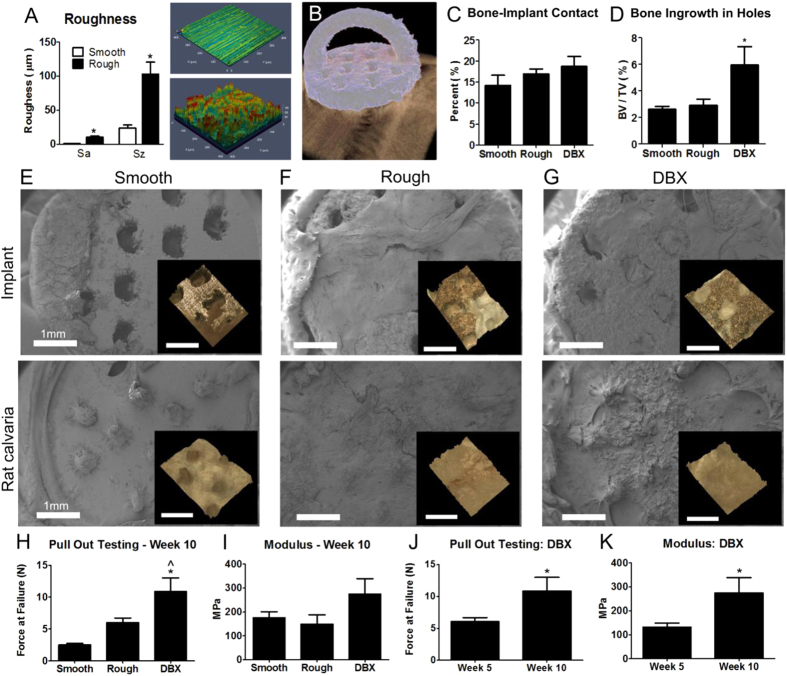
The effect of surface roughness and demineralized bone matrix on bone growth and mechanical properties of new bone growth into porous disks. Laser sintered implants were manufactured and polished or treated by blasting and acid etching to produce smooth and rough surfaces for calvarial implantation in rats. Laser confocal microscopy confirmed reduced surface roughness and peak to valley heights (**A**) of smooth surfaces (**A** top right) in comparison to rough surfaces (**A** bottom right). MicroCT image analysis (**B**) was conducted of calvarial implants after 10 weeks to assess bone-to-implant contact (**C**) and bone ingrowth in holes (**D**). SEM micrographs after pull-out testing of the implant ((**E–G**) top, with inset optical images) and calvaria ((**E–G**) bottom, with inset optical images) showed bone growth and attachment to implants. Pull out testing revealed force at failure (**H**) and modulus (**I**) 10 weeks after implantation. Force at failure for pull out testing (**J**) and modulus (**K**) was also compared at 5 and 10 weeks for the DBX treated group. One-way ANOVA with Bonferroni correction, p < 0.05, * vs. smooth or week 5, ^ vs. rough.

To further analyze the effects of surface roughness and DBX-treatment on implant osseointegration, mechanical testing was conducted. For these experiments, the laser sintered disks were treated by grit blasting and acid etching to have the microscale and nanoscale roughness as well as the hydrophilicity described above. A second set of disks was polished, producing a smooth surface. Unlike all other disks and implants used in this study, which were sterilized by gamma radiation, the polished disks were sterilized by autoclaving. Laser confocal analysis revealed significantly higher surface roughness and peak-to-valley height on rough surfaces compared to smooth surfaces (Fig. 3A). Smooth or rough implants were produced with an arch for mechanical testing (Fig. 3B). Smooth, rough or rough implants treated with DBX were placed on athymic nude rat calvaria, and microCT analysis was performed at 10 weeks.

Although bone-to-implant contact (Fig. 3C) was not significantly different across groups, bone in-growth in implant holes (Fig. 3D) was significantly increased for rough implants with DBX compared to smooth and rough implants alone. Electron and optical images of smooth implants (Fig. 3E), rough implants (Fig. 3F) and rough implants used with DBX (Fig. 3G) (top) and calvaria (bottom) after mechanical testing at 10 weeks revealed bone ingrowth into implants, with more bone observed on the rough implants regardless of DBX use compared to smooth implants. Rat calvaria with smooth implants showed bony protrusions (Fig. 3E, bottom) that were retained after implant pull-out testing, while protrusions on calvaria with rough implants with and without DBX (Fig. 3F, G bottom) were partially removed with the implant as part of mechanical testing. Force at failure of rough implants was significantly higher when DBX was included than non-treated smooth or rough implants at 10 weeks (Fig. 3H). Although the average modulus was higher for DBX-treated rough implants, no significant differences were found among groups (Fig. 3F). Pull-out testing was performed for rough implants with DBX at 5 and 10 weeks, showing higher force at failure (Fig. 3J) and modulus (Fig. 3K) at 10 weeks compared to 5 weeks.

### Custom-fit wrap implants osseointegrated with rabbit tibias

**Figure 4:**
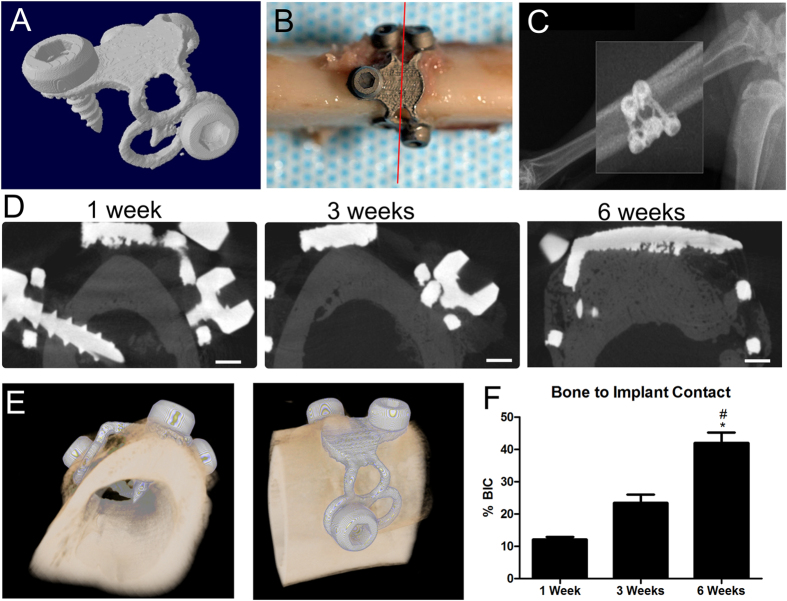
MicroCT analysis of endosteal wrap implants shows bone growth over 6 weeks. Wrap implants were designed and manufactured (**A**) for implantation around rabbit tibias ((**B**), with the red line indicating cross sections for microCT analysis). MicroCT scans of the entire implant (**C**) and cross sections were taken after 1, 3, and 6 weeks after implantation (**D**). Reconstructed scans (**E**) were used for analysis of bone-to-implant contact (**F**). Scale bars represent 1 mm. One-way ANOVA was performed with Bonferroni’s correction, p < 0.05, *vs. 1 week, # vs. 3 weeks.

**Figure 5:**
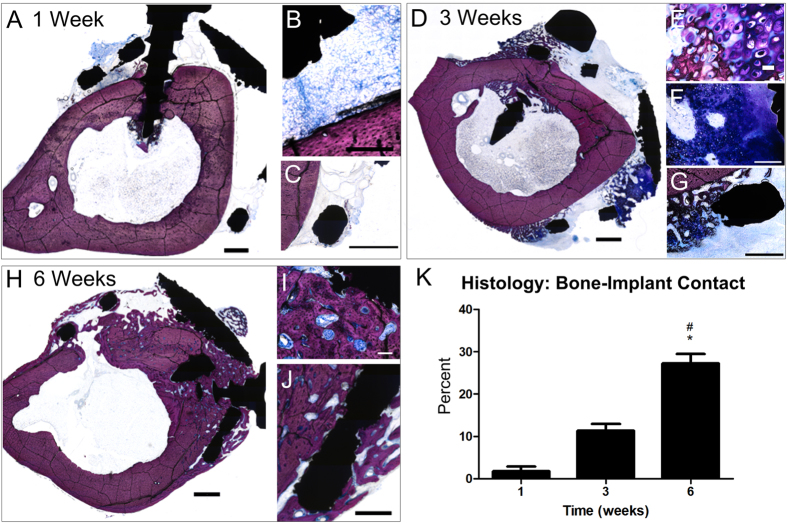
Bone growth into and around endosteal wrap implants on rabbit tibias. Histological sections of wrap implants stained with Stevenel’s Blue after 1 week ((**A)**, with enlarged (**B)** showing bone, and (**C)** showing connective tissue), 3 weeks ((**D**), with enlarged (**E,F)** showing cartilage and (**G**) showing woven bone), 6 weeks ((**H**), with enlarged (**I**) showing bone in contact with inside of implant and (**J**) showing new bone around outside of implant) and used to analyze bone-to-implant contact (**K**). Scale bars for (**A–D,H**) represent 1 mm, scale bars for (**G**) and (**I**) represent 500 um, scale bar for F represents 200 um, scale bar for (**J)** represents 100 um and scale bar for (**E)** represents 20 um. For histomorphometric analysis, one-way ANOVA was performed with Bonferroni’s correction, p < 0.05, *vs. 1 week, # vs. 3 weeks.

To demonstrate structural implant functionality, we analyzed osseointegration of custom-manufactured wrap implants placed around rabbit tibias. Because previous experiments in this study showed osseointegration without the use of DBX, we did not use any bone graft substitutes in this experiment in order to focus on the implant geometry. We were also able to achieve bone growth through the implant holes even without the presence of DBX, suggesting that the surface modification alone would be successful in supporting osseointegration of an implant geometry that was more clinically relevant in a larger animal model. Laser sintered wrap implants were manufactured to fit snugly around tibias of New Zealand White rabbits (Fig. 4A). Implants were secured with four screws that penetrated into the bone marrow cavity, and microCT cross sections of the entire implant were taken for analysis of osseointegration (Fig. 4B,C). MicroCT images at 1, 3 and 6 weeks showed continuous bone growth filling the void space between the tibia and the implant by 3 weeks, and even expanding beyond the implant at 6 weeks (Fig. 4D). MicroCT 3D reconstructions were able to provide better representative images of implants around tibias at 6 weeks (Fig. 4E). Quantitative analysis revealed significantly higher bone-to-implant contact values at 6 weeks compared to at 1 and 3 weeks (Fig. 4F). Histological sections of implants provided a more detailed view of bone formation (Fig 4, S3). At 1 week, there were small gaps remaining between the implant screws and bone (Fig. 5A), with new bone (Fig. 5B) and connective tissue (Fig. 5C) forming. At 3 weeks, further bone growth was achieved (Fig. 5D), with cartilage (Fig. 5E,F) and woven bone prominent near the implant (Fig. 5G). At 6 weeks, fully formed bone was present (Fig. 5H) in contact with the inside of the implant (Fig. 5I), with bone formation occurring around the outside of the implant as well (Fig. 5J). Bone-to-implant contact analyzed from histological images showed significantly higher values at 6 weeks compared to at 1 and 3 weeks (Fig. 5K).

### Wrap implants were customized for dental implant placement in patients

**Figure 6:**
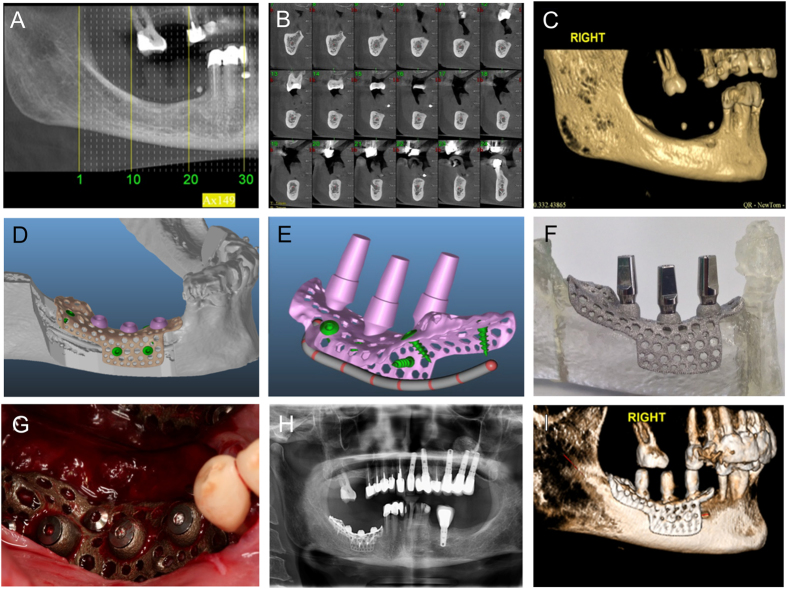
A patient customized endosteal implant was created to enhance bone regeneration for future dental implant placement. A CT scan was taken of the patient (**A**) to plan implant placement (**B, C**). A customized Ti-6Al-4 V implant was designed in software (**D, E**), with purple representing implant posts and green representing stabilizing screws. The implant was manufactured as one piece (**F**) and implanted in the patient (**G**). A follow-up panoramic X-ray was taken to evaluate osseointegration and the bone to implant contact after three months (**H**), and a CT scan performed after eight months (**I**).

To translate our animal studies clinically, we developed patient-specific wrap implants to induce bone regeneration with a one-step surgical procedure under a Helsinki Committee-approved protocol. Two case studies are described (Fig. 6 and Fig. S4). CT scans were taken of the patient at the site of intended implant placement (Fig. 6A), and reconstructed scans (Fig. 6B,C) were used to develop a custom, porous endosteal wrap implant (Fig. 6D) using the same laser sintering parameters and implant surface treatments as described for the disks and wrap implants above. One-piece implants included a porous base similar to the pore geometry used in rat calvaria studies, stabilizing screws as used in the rabbit studies, and implant posts to be used for eventual crown placement (Fig. 6E). Sintered implants were fit to an additively manufactured plastic mold of the patient’s mandible (Fig. 6F) to ensure correct placement. Prior to perforating the bone, the implants were placed to ensure that they fit the placement site. The implants were then removed and the bone was perforated using a dental bur. The site was coated with DBX, and the implant placed and secured to the patient’s mandible at the predetermined location (Fig. 6G). The secured implant was coated with DBX and the flap was passively closed. A panoramic X-ray was taken at 3 months post-surgery (Fig. 6H), and a CT scan was taken at 8 months post-surgery and 6 months post-loading (Fig. 6I). At 8 months, the implant was osseointegrated with continued functional loading and no complications reported by the patient. The implant was loaded and in function; no pain or infection was reported. The second case in which the patient received implants on both sides of the mandible is presented in Fig. S4.

## Discussion

Similar to how computer aided design/computer aided manufacturing (CAD/CAM) revolutionized solid implant fabrication years ago, additive manufacturing technology is impacting the field by making the promise of precision medicine accessible to patients requiring complex reconstructive surgery^20^,^21^. Here, we show that 3D Ti-6Al-4 V implant surfaces can be designed that enhance osteoblast response *in vitro* and osseointegration *in vivo* compared to smooth surfaces, and that these additively manufactured and processed surfaces can be combined with DBX for osseointegration beyond the bone envelope *in vivo*. This conclusion was achieved in a stepwise process that included surface modification at the microscale and nanoscale, validation that the modified surface supported osteoblastic differentiation of normal human osteoblasts; *in vivo* demonstration that the surface modification was sufficient to support bone formation using qualitative and quantitative imaging and biomechanical parameters, translation of the technology to a more challenging animal model, and finally use of the technology to support implant placement in two edentulous human patients with severe bone loss.

This study has shown that the implant surface can influence biological response even without the addition of exogenous factors. Surface roughness at multiple scales is necessary for increasing osteoblast response and osseointegration^7^,^13^,^22^,^23^. While we did include a polished smooth surface in our initial studies to verify the superiority of implant surfaces with micro-/nano-roughness, we chose to focus on rough surfaces in our rabbit and clinical studies as they are more clinically relevant. Although we did not polish inside the implant holes, these are not the first site of contact with the calvarial bone. We believe that the large surface area of the implant underside compared to the side wall of the holes contributed more to the mechanical testing and osseointegration. In addition, this is the surface along which we measured the bone to implant contact.

In addition to the micro-texture and nano-texture of the surface, other surface parameters such as wettability have been shown to influence the biological response^24^. Dental implants with topographies similar to those used in this study that have retained a hydrophilic surface exhibit more rapid osseointegration than implants that are hydrophobic^25^. We did not directly compare hydrophilic implants to hydrophobic implants with identical surface morphology. However, it has been shown in other studies that autoclave sterilization results in a hydrophobic surface^26^,^27^. Because we autoclave sterilized the polished disks used in the *in vivo* study, our mechanical testing experiments were only able to assess the combined effects of surface topography and hydrophilicity on cell response. Whether the relative hydrophilicity or hydrophobicity of the surface contributed in a significant way to the differences in osseointegration between the disks with smooth and rough surfaces is not known.

MicroCT evaluation of bone-to-implant contact provides a 3D analysis of implant osseointegration and new bone infiltration into the implant pores. Total implant analysis cannot be achieved with conventional histology, which evaluates osseointegration only at one cross sectional location. Previous work analyzing bone ingrowth into porous titanium implants indicated that depending on pore size and location, new bone did not form in the same manner across all pores^28^,^29^,^30^, which helps explain why our bone-to-implant values achieved through microCT are different from those achieved via histomorphometry. However, the disadvantage of microCT evaluation lies in its inability to visualize non-mineralized tissue, which is easily identifiable through histological sectioning and staining^31^. Interference from the titanium can also introduce scatter and lower resolution in the image. Therefore, both methods of analysis are valuable and can contribute to evaluation of osseointegration.

In this study, we used two different animal models to focus on the scientific and clinical aspects of bone regeneration and osseointegration. We used rats to develop our surgical procedure and evaluate implant osseointegration. Before transitioning to human clinical trials, we needed to evaluate feasibility of structurally similar constructs in a higher order animal model. We chose the rabbit tibia both as having a more geometrically comparable curvature to the human mandible compared to the rat calvaria, as well as an alternate way of showing osseointegration in the long bone. Because human maxillofacial regeneration is composed of both endochondral and intramembranous ossification, we wanted to verify endochondral bone formation in the rabbit tibia in response to the implant after observing successful intramembranous bone formation in our rat cranial onlay model.

One potential design concern for these one-piece custom implants is roughness at the abutment connection. Traditional dental implants leave this area polished to prevent bacterial colonization and subsequent peri-implantitis^32^. Studies have shown an altered bacterial attachment profile on titanium and Ti-6Al-4 V rough surfaces compared to smooth surfaces, but it is still unclear how this may affect clinical response after implantation^33^,^34^,^35^. In our study at 8 months post-implantation, no complications or bacterial contamination were noted in patients. However, future studies may wish to identify post-processing methods that are able to differentially treat portions of the implant to achieve spatially disparate smooth and rough surfaces.

Although bone grafts and bone graft substitutes are commonly used in ridge augmentation, cases that require substantial vertical regeneration of bone are not predictably successful. These patients require multiple procedures and the regeneration strategy and treatment plan vary considerably from case to case^36^. Our results, achieved through iterative *in vitro* and *in vivo* studies, suggest that additive manufacturing can be used successfully to produce implants that meet the demands of precision medicine by tailoring the shape to the individual patient’s needs. The use of DBX had an osteoinductive effect, enhancing osseointegration of the roughened surface in the rat calvaria. Based on these results, we continued to use DBX with human implantation, and suggest this as a best practice for future clinical use. The combination of surface topography modification of laser sintered Ti-6Al-4 V at the microscale and nanoscale with clinical best practices can lead to osteogenesis and ultimately osseointegration of implants, even for patients with limited bone, enabling restoration of form and function. Though this study only indicates one clinical application of additive manufacturing for dentistry, we hope that others will apply our findings to bone regeneration in facial reconstruction after trauma, cancer or in other compromised cases.

## Methods

### Study Design

The objective of this study was to evaluate the *in vitro, in vivo* and human clinical performance of surfaces and implants manufactured using laser sintering with post-processing surface treatment. Our hypotheses were that laser sintered surfaces with hierarchical surface roughness could enhance osteoblast response *in vitro* and increase osseointegration *in vivo* compared to smooth surfaces and implants. We also hypothesized that use of DBX with the implant would increase osseointegration in a rat calvaria model, and that these results would translate to rabbit and human implantation. All cell culture studies were repeated at least three times to ensure repeatability of data. Power analysis was conducted for each animal study, and animals were assigned randomly to experimental groups during surgery. Severe behavioral and physical changes in animals were considered as humane endpoints upon which to end data collection. Blind analysis was conducted for histomorphometric and microCT evaluation of bone-to-implant contact. Significance for all studies was determined using one-way analysis of variance (ANOVA) with Bonferroni’s Multiple Comparison Test and a p value of less than 0.05. Outliers were determined and excluded based on Grubbs’ extreme studentized deviate test.

### Material Manufacturing

Solid disks, porous calvarial implants and tibial wrap implants and clinical implants were produced by laser sintering (EOS GmbH, Krailling, Germany). Ti-6Al-4 V powder particles 25-45 μm in diameter (Advanced Powders & Coatings, Quebec, Canada) were sintered using a Ytterbium fiber laser with a scanning speed of 7 m s−1, 1054 nm wavelength, 200 W continuous power and 0.1 mm spot size. Solid disks used for *in vitro* studies were 15 mm in diameter and 1 mm in height. Smooth disks and implants were polished with aluminum oxide sanding paper (P240, Norton Abrasives, Paris, France) and referred to as LST-M for solid surfaces or Smooth for porous implants. Rough disks and implants were blasted with calcium phosphate particles in a proprietary manner, then then acid etched for 90 minutes in 10% of a 1:1 ratio of maleic and oxalic acids. All materials were sterilized by gamma radiation prior to cell culture or implantation unless otherwise stated.

### Material Characterization

Scanning electron microscopy (SEM, Zeiss Ultra60 FE-SEM, Oberkochen, Germany) was used to visualize surfaces, implants, and calvaria. Calvaria and implants with biological material were dehydrated in a series of ethanol: at least two hours in 15%, 30% and 45% ethanol, at least one hour in 60%, 75%, 90% and twice in 100% ethanol, 1:1 exchange in 100% ethanol and hexamethyldisilazane (HMDS), and twice for 30 minutes in HMDS. SEM was conducted with an accelerating voltage of 4 kV and working distance of 4 mm for material and 10 mm for biological samples. Before imaging, biological samples were platinum sputtered for 90 seconds at 35 μA. Smooth control disks were analyzed with confocal microscopy (Zeiss LSM 710, Jena, Germany) and scanning electron microscopy to ensure that the surfaces were significantly less rough than the test disks. Average roughness (S_a_) and peak-valley height (S_z_) measurements were conducted under 20 × magnification (Plan-Apochromat 20 × /0.8 M27 objective) over a 425 μm × 425 μm area z-stack with a step size of 5 μm.

### Cell Response

Normal human osteoblasts (NHOst cells, Lonza, Basel, Switzerland) were seeded onto disks at a density of 10,000 cells per cm^2^ according to tissue culture polystyrene (TCPS) surface area, or 20,000 cells per well in a 24 well plate. Cells were fed with full medium (DMEM supplemented with 10% fetal bovine serum, 1% penicillin/streptomycin) 24 hours after plating, then every 48 hours until reaching confluence on TCPS. Cells were fed at confluence on TCPS and harvested 24 hours afterward for biological analysis. At harvest, medium was aliquoted and stored to analyze osteocalcin (BT-480, Alfa Aesar, Ward Hill, Massachusetts), osteoprotegerin (DY805, R&D Systems, Minneapolis, Minnesota), vascular endothelial growth factor (VEGF, DY293B, R&D Systems) and bone morphogenetic protein 2 (BMP2, 900-K255, PeproTech, Rocky Hill, New Jersey) levels. Cell layers were rinsed twice in phosphate buffered saline and then stored in 0.05% Triton X-100 at −20 °C overnight, sonicated and analyzed for DNA content (E2670, Promega, Madison, Wisconsin) and protein (23225, Life Technologies, Carlsbad, California). Alkaline phosphatase specific activity was analyzed by cleavage of *p*- nitrophenol from *p*-nitrophenylphosphate at pH 10.2. These methods are described in greater detail in references^12^,^17^,^37^.

### Rat Calvaria Experiments

5.0 mm disks with 12 evenly spaced 0.5 mm holes were generated via additive manufacturing by laser sintering Ti-6Al-4V alloy powder. The resulting disks were grit blasted and then acid-etched to create a specific micro-surface as described previously^17^. Disks were subjected to a cleaning protocol^17^ and then sterilized via gamma radiation. Three studies were conducted using a surgical protocol where disks were implanted subperiosteally on the surface of the calvarial bone of 250–300 g 8 week old male Sprague-Dawley rats (Harlan Laboratories, Indianapolis, Indiana) or athymic (RNU/RNU) nude rats (Charles River, Wilmington, Massachusetts). All animal procedures were conducted with the institutional approval of Virginia Commonwealth University (Richmond, VA). Following the elevation of a periosteal flap, multiple 0.3 mm holes were drilled through the cortical bone into the marrow space of the left parietal bone (15 to 20 holes per calvarium). The disks were placed over the holes, and the flap was sutured over the disk.

To determine the effect of pretreating the implantation site via *in situ* decalcification, exposed bone around the holes was left untreated (N = 6) or treated with 24% EDTA for five minutes (N = 6). A third group of rats was treated with 37% phosphoric acid for 1 minute prior to drilling the holes (N = 6). Animals were euthanized after 5 and 10 weeks.

To determine the osteogenic capability of the disks, calvaria were not treated with either decalcification protocol prior to placing the disks. Disks were then implanted with and without the addition of demineralized bone matrix putty (DBX^®^, donated by the Musculoskeletal Transplant Foundation, Edison, NJ) (N = 7 male athymic nude rats per group, Charles River). DBX was applied to the bone surface and the top of the disk prior to closing the periosteum. Animals were euthanized at 15, 35, and 70 days, denoted as 2, 5, and 10 weeks, respectively. Samples in both studies were analyzed by microCT and ground histology sections stained with Stevenel’s Blue to determine the bone-to-implant contact and bone ingrowth into the holes of the disk via histomorphometry.

Osseointegration was also characterized as a function of the pull-out strength of the implants at 5 and 10 weeks. Implants were fabricated as above, but a handle was incorporated into the design to interface with the mechanical testing apparatus. A second set of implants was produced by additive manufacturing but the surface was polished rather than grit blasted and acid etched. Male athymic nude rats were used for each treatment group (N = 8). Implants were placed using the DBX protocol. The periosteum was closed with a purse string suture, leaving the arch exposed for mechanical testing. Mechanical testing was performed at harvest as described below.

### Rabbit Experiment

Male New Zealand White rabbits (N = 30, 3.0 to 3.2 kg each) were weighed and anesthetized with 2% isofluorane in an anesthetic chamber, then isofluorane was continually administered for the duration of the surgery via a nose mask. 50 mg/kg ketamine and 5 mg/kg xylazine were administered to sedate the rabbit. The left leg was shaved and disinfected with ethanol and chlorhexidine. The tibia was exposed and four pilot holes were drilled for placement of the implant screws. Drilling was conducted at low speed, intermittently and under a continuous stream of saline that cut through the bone fragments and cooled the drilling area. The implant was placed on the tibia and secured with screws that penetrated into the bone marrow cavity. After placing the implant, the subcutaneous tissue and skin were closed with absorbable surgical sutures. The isoflurane was removed, and the rabbit was allowed to awaken and recover on a heating pad. Rabbits were given 0.01 mg/kg buprenorphine twice daily for 3 days following surgery, and 20 mg/kg Ceporex antibiotic 7 days following surgery. Health, weight, food intake and wound state were evaluated twice daily during the first 6 days after surgery to ensure animal recovery. Rabbits were examined daily for the remaining recovery period. At 1 (N = 3), 3 (N = 16) and 6 (N = 11) weeks, rabbits were anesthetized with 2% isoflurane. The surgical site was re-opened to retrieve fragments of bone containing the implants. The animals were then euthanized by air embolism with a 5–50 ml/kg intravenous injection of air. Rabbit experiments were carried out at the University Lodz, approved by the Animal Ethics Committee at the University of Lodz and carried out in accordance with approved guidelines.

### MicroCT

All scans were conducted on a Bruker Skyscan 1173 MicroCT, hardware version A. Images were scanned using Skyscan Control Software version 1.6 (Kontich, Belgium). Calvaria were scanned at a resolution of 2240 × 2240 pixels (image pixel size of 14.74 μm), with x-rays of 105 kV and 65 μA using a 0.25 mm brass filter and exposure time of 500 ms and a rotation step of 0.4°. When assessing the requirement for DBX, calvaria were scanned as above using 130 kV, 61 μA, exposure time of 1600 ms, and an image pixel size of 11.86 μm. All calvarial implants that were not destined for mechanical testing were fixed in 10% neutral buffered formalin prior to scanning. Those calvarial implants intended for mechanical testing were scanned as fresh tissue. Rabbit tibial implants were scanned at a resolution of 1120 × 1120 pixels, 75 Kv, 106 μA, exposure time of 1200 ms, using a 0.25 mm brass filter with an image pixel size of 20.85 μm over 360° and a rotation step of 0.400°. Samples were fixed in 10% neutral buffered formalin prior to scanning. A standard Feldkamp reconstruction was performed on a subset of samples using NRecon Software version 1.6.9.17 (Kontich, Belgium) with a Gaussian smoothing kernel of zero and a beam hardening correction of 20%. Analysis of all samples was conducted with CTAn software version 1.14.4.1 (Kontich, Belgium). Bone-to-implant contact was calculated as a function of the total bone volume immediately connected to the total volume of the implant in all studies. Bone ingrowth into calvarial disk holes was calculated as a function of total bone volume within the volume of all of the disk holes of the implant.

### Histology

Samples from all studies were commercially processed (Histion, Everett, WA). Briefly, samples were embedded in methyl methacrylate and one ground section was taken from each specimen, which, if possible, passed through an axis of the disk containing four holes (for rat calvaria experiments). Sections were stained with Stevenel’s blue/van Gieson^38^. Samples were photographed using Zen 2012 Blue Edition software with an AxioCam MRc5 camera and Axio Observer Z.1microscope (Carl Zeiss Microscopy, Oberkochen, Germany). Bone-to-implant contact (BIC) in both the calvarial disk studies as well as the tibial implant study was calculated by dividing the bone contact perimeter by the total perimeter of the implant. Bone ingrowth into calvarial disk holes was calculated by dividing the bone area by the total area of each of the four holes of a sample.

### Mechanical Testing

To quantitatively assess osseointegration of calvarial disks, pull out force testing was conducted using a 30 kN load cell for N = 8 animals per group. Ti-6Al-4 V laser sintered disks with a mounting bracket were manufactured and processed in the same way as described for surfaces used for cell culture and histological analysis. Smooth controls were prepared by sanding disks with aluminum oxide 240 grit sanding sheets (Norton Saint-Gobain, P240) and autoclaved at 121 °C for 30 minutes. Smooth control implants were examined by SEM and confocal microscopy and found to be significantly less rough compared to normal implants. Surgical implantation was performed as described for the other calvarial implants, but the periosteum was closed around the base of the mechanical testing bracket.

### Clinical Cases

All clinical procedures were approved by the Helsinki Committee of Tel Aviv University and the Ministry of Health of Israel and carried out in accordance with approved guidelines. Informed consent was obtained from all subjects. Two patients were screened and selected as candidates for custom wrap implant placement. For these patients, all currently known and accepted methods to rehabilitate edentulous posterior mandibles, including partial dentures, were found to be inappropriate. Initial evaluation of the implant site was performed by cone beam computed tomography (cbCT). Custom subperiosteal jaw wraps with implant posts were designed using the AB software program (AB Dental, Ashdod, Israel). Implants were fabricated by laser sintering in the same manner as described previously for implants produced for culture and animal studies, and gamma irradiated prior to implantation. Local anesthesia was administered for the surgical procedure. A crestal incision was made in the mandible, and the full flap was elevated. After examining the implant fit to the mandibular bone, bur holes were drilled along the coronal aspect and the side of the mandible to expose the bone marrow. A layer of DBX was placed on the bone surface before implant placement. The implant was placed on top of the mandibular crest as previously planned and secured with four miniscrews. Another layer of DBX was placed on top of the implant prior to closure. After placement, the gingiva was closed passively with two horizontal mattress sutures (Vicryl), and with interrupted sutures. Patients were prescribed antibiotics and local analgesics, and a final panoramic X-Ray scan was conducted to verify implant placement. The patients were followed for 3 months with no complications noted. At 3 months post-surgery, a second panoramic X-Ray was taken and the implants were loaded. A final follow-up CT scan was taken of patients after 8 months to observe osseointegration and implant function.

## Additional Information

**How to cite this article**: Cohen, D. J. *et al.* Novel Osteogenic Ti6Al4V Device for Restoration of Dental Function in Patients with Large Bone Deficiencies: Design, Development and Implementation. *Sci. Rep.* **6**, 20493; doi: 10.1038/srep20493 (2016).

## Supplementary Material

Supplementary Information
