# Supplementary material for: Novel Osteogenic Ti-6Al-4V Device For Restoration Of Dental Function In Patients With Large Bone Deficiencies: Design, Development And Implementation
Source: Sci Rep. 2016 Feb 8;6:20493. doi: 10.1038/srep20493 (PMC4745084; doi:10.1038/srep20493)
Supplement: Supplementary Information [file srep20493-s1.pdf]

Novel Osteogenic Ti_6Al_4V Device for Restoration of Dental Function in Patients with Large Bone Deficiencies: Design, Development and Implementation

Authors: D. J. Cohen, A. Cheng, A. Kahn, M. Aviram, A. J. Whitehead, S. L. Hyzy, R. M. Clohessy, B. D. Boyan, Z. Schwartz

SUPPLEMENTAL INFORMATION

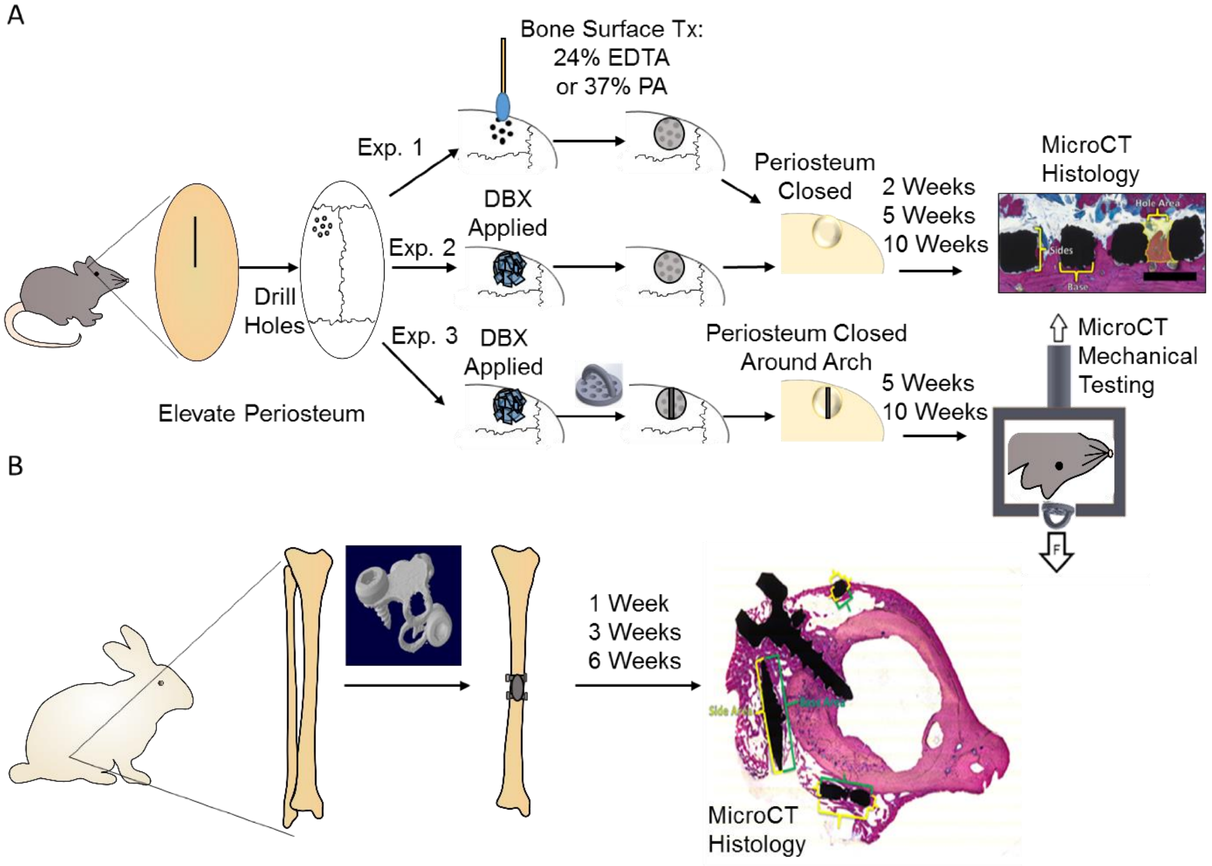

Fig. S1. Schematic of all animal experiments. The first set of experiments was conducted on rat calvaria to assess the effect of surface treatment of calvaria on implant osseointegration (A top), the second was to evaluate the effect of roughness on implant osseointegration with or without DBX (A middle) and the third was to analyze mechanical strength of osseointegrated implants with or without DBX (A bottom). Further in vivo work was conducted in a rabbit tibial model with a more clinically representative wrap implant to assess implant osteogenesis (B).

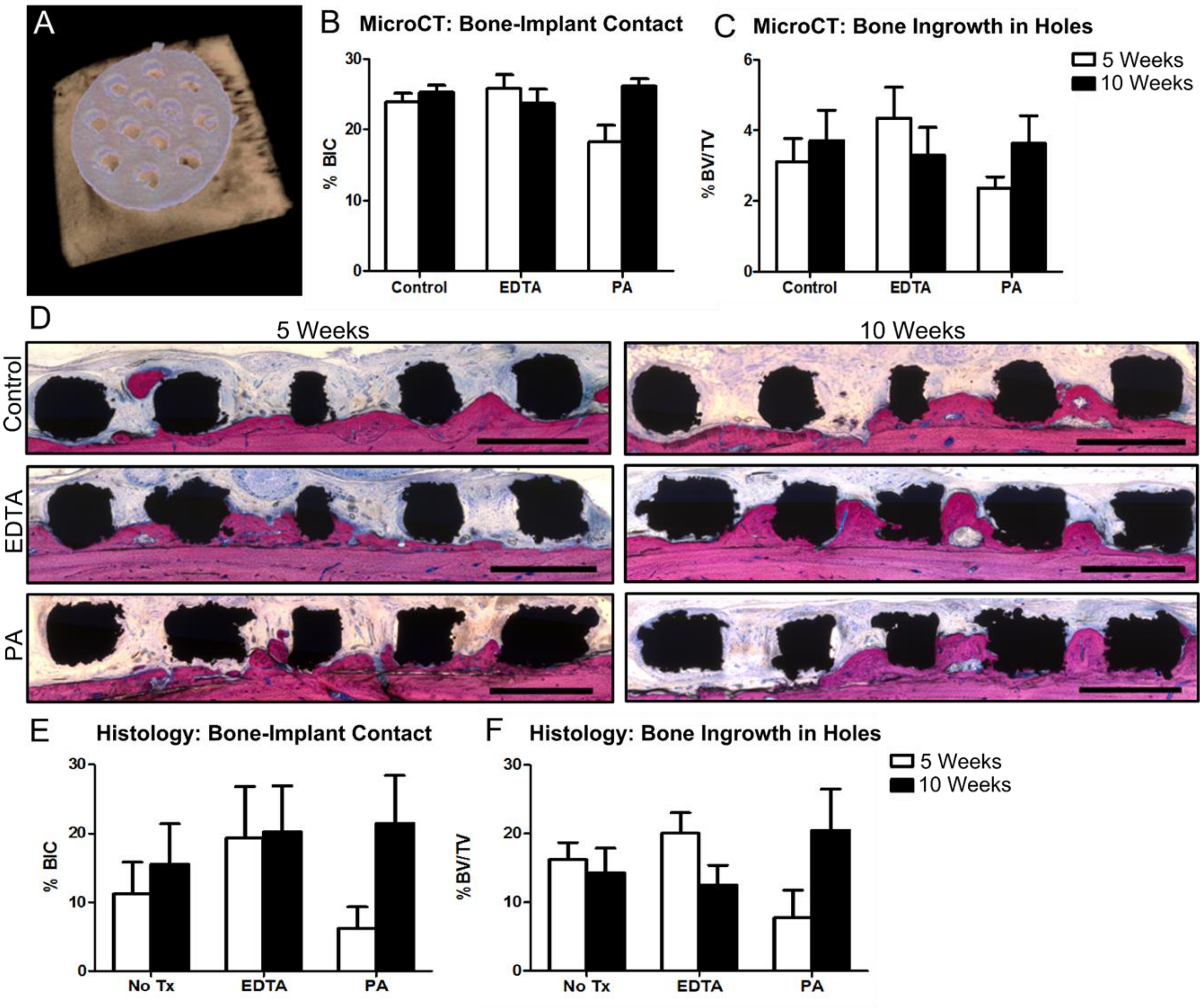

Fig. S2. The effect of pre-treatment on calvarial bone growth into porous disks. Disks were implanted on Sprague-Dawley rat calvaria with no treatment (control), EDTA, or phosphoric acid treatment prior to implantation, and bone-to-implant contact was assessed after 5 and 10 weeks. MicroCT was used to visualize disks on calvaria (A), bone-to-implant contact (B) and bone ingrowth in holes (C) was assessed in implant cross sections of the total implant. Histological sections were taken after 5 (D left) and 10 weeks (D right) for each group, and bone-to-implant contact (E) and bone ingrowth in holes (F) was measured by histomorphometry. Scale bar for histological images represents 1mm. Values were not statistically significant across treatment groups.

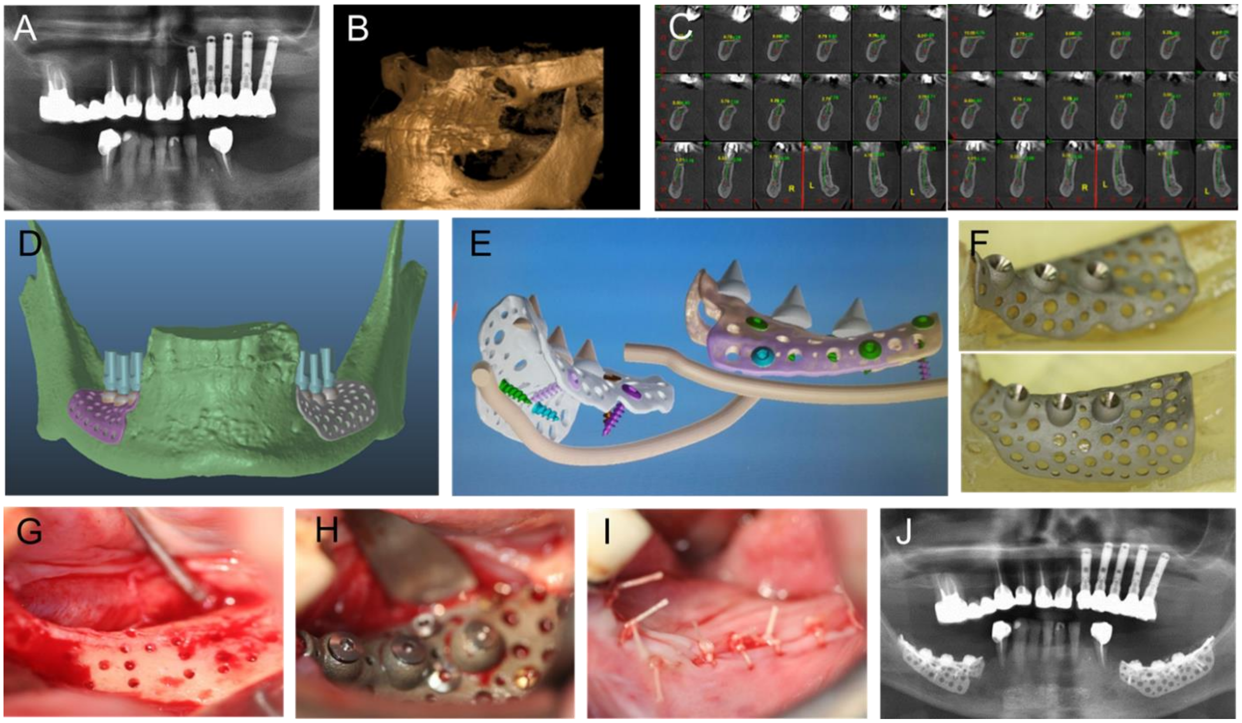

Fig. S3. A second case of a patient customized endosteal implant. A CT scan was taken of the patient (A) to plan implant placement (B, C). Customized Ti6Al4V implants were designed using software (D, E). The implants were manufactured as one piece for each side. For each implant, small holes were drilled into the patient's jaw prior to implant placement to ensure exposure to stem cells and progenitor cells (G), the implant was placed and stabilized (H) and the gum and periosteum were sutured over the implant (I). A follow-up panoramic X-ray was taken to evaluate osseointegration and the bone to implant contact after three months (J).

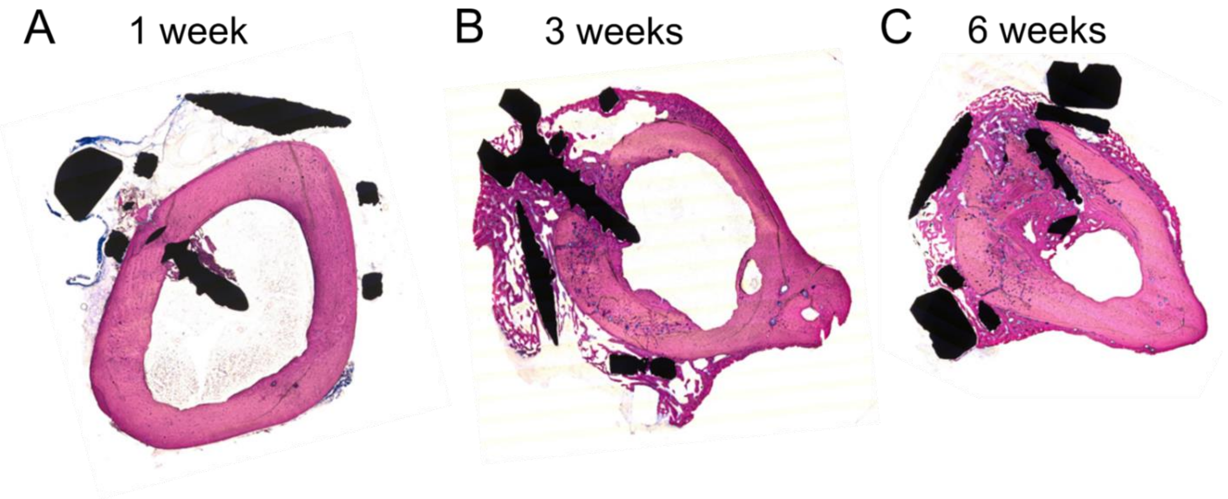

Fig. S4. Bone growth into and around endosteal wrap implants on rabbit tibias. Histological sections of wrap implants stained with Stevenel's Blue at 1 week (A), 3 weeks (B), 6 weeks (C).
